# Longitudinal variance-components analysis of the Framingham Heart Study data

**DOI:** 10.1186/1471-2156-4-S1-S22

**Published:** 2003-12-31

**Authors:** Stuart Macgregor, Sara A Knott, Ian White, Peter M Visscher

**Affiliations:** 1 Biostatistics and Bioinformatics Unit, University of Wales College of Medicine, Heath Hospital, Cardiff, United Kingdom; 2 Institute of Cell, Animal and Population Biology, Ashworth Laboratories, The University of Edinburgh, United Kingdom

## Abstract

The Framingham Heart Study offspring cohort, a complex data set with irregularly spaced longitudinal phenotype data, was made available as part of Genetic Analysis Workshop 13. To allow an analysis of all of the data simultaneously, a mixed-model- based random-regression (RR) approach was used. The RR accounted for the variation in genetic effects (including marker-specific quantitative trait locus (QTL) effects) across time by fitting polynomials of age. The use of a mixed model allowed both fixed (such as sex) and random (such as familial environment) effects to be accounted for appropriately. Using this method we performed a QTL analysis of all of the available adult phenotype data (26,106 phenotypic records).

In addition to RR, conventional univariate variance component techniques were applied. The traits of interest were BMI, HDLC, total cholesterol, and height. The longitudinal method allowed the characterization of the change in QTL effects with aging. A QTL affecting BMI was shown to act mainly at early ages.

## Background

In this paper we analyze the Framingham Heart Study offspring data using univariate and multivariate variance component techniques, with particular emphasis on how inherited factors related to heart disease change over the life of an individual.

### Data available

There were 4692 individuals in the study. The data were ascertained in two cohorts. The first had up to 21 trait measures for the 40 years following 1948. The second cohort had up to 5 trait measures for the 20 years following 1971. Genotype data were available for 1702 individuals. The vast majority of individuals in the study had all their measures when they were age 20 or older; measures at younger ages were not analyzed. Phenotype data was available for 2885 individuals. In total, 26,106 phenotypic records were used in the full multivariate analysis. The traits considered were body mass index (BMI), height, fasting high density lipoprotein cholesterol (HDLC), and total cholesterol.

### Manipulation of data for analysis

The data were reorganized to associate a record with an age rather than an examination number. Ages ranged from 20 to 95. For the initial analyses the data were split into six age bands; the bandings were trait at ages 20 to 30 (age nearest 30 used), trait at ages 30 to 40 ... 70 to 80. The number of individuals with at least one record in the relevant age band is shown in Table 1. When an individual had two or more records in a given decade, only the latter of these measures was included. In addition, we created one large band with a single measure on an individual between the ages of 40 and 60 (age nearest 60 used, denoted the '40–60' band). This band facilitated a single univariate analyses of most of the individuals (up to 2560).

## Methods

### Univariate analyses

For BMI and height, potential covariates were sex, cohort, cigarette consumption, and alcohol consumption. For HDLC and total cholesterol, BMI and an indicator variable for hypertension treatment were also considered.

#### Polygenic

The traits were examined for variation across time using Residual Maximum Likelihood (REML, program ASREML) [1] to calculate polygenic heritabilities in the six age bands.

#### Quantitative Trait Locus (QTL)

Standard univariate variance components (VC) analyses were done using the SOLAR program [2] and confirmed using ASREML. LODs were calculated using multipoint IBDs (identity by descent coefficients) every 1 cM.

### Longitudinal Analysis

#### Polygenic

A RR model was fitted to the full (up to 26,106 records) data set for each trait. The model allowed both the additive genetic effect and the permanent environment term to vary linearly with age. The model was therefore

y_ij _= μ + (a_i1 _+ a_i2 _× age*) + (c_i1 _+ c_i2 _× age*) + f_i _+ e_ij_,

where y_ij _is the phenotype of individual i at time point j, μ represents the fixed effects, e_ij _is the special or temporary environmental effect, f_i _is an effect for family or household and the terms a_i1_, a_i2_, c_i1_, and c_i2 _are the coefficients of the linear polynomial linking mean corrected age (age*) to the relevant genetic and permanent environmental terms. Note that using age* instead of age means the polynomials are *orthogonal *(see [3]). The genetic and permanent environment terms were assumed to have unstructured variance-covariance matrices, denoted by matrices **G **(with entries g_ij_) and **P **(with entries p_ij_), respectively. These estimated (co)variances are then linked to a relevant set of n ages (in this case 20–95). For example, for the genetic effect at age x the variance contribution is

g_11 _+ 2 × [x - mean(x)] × g_12 _+ [x- mean(x)]^2 ^× g_22_.     (1)

In matrix notation the n × n matrix, **T**, of phenotypic (co)variances is hence decomposed as

**T **= **XGX**^T ^+ **XPX**^T ^+ σ_e_^2^**I**,     (2)

where **X **= (1 age*) with **1 **an n-vector of 1s and age* a vector of ages from age*(1) to age*(n). σ_e _^2 ^is the e_ij _term variance and **I **is the identity matrix. In cases where a family effect is included, an additional term, σ_f_^2^**11**^T^, where σ_f_^2 ^is the variance term associated with the family effect, should be added to equation (2) (assuming no relationship between age and family effect).

Estimates of the phenotypic and component variances (genetic, permanent environment, error) at any age are given by the appropriate diagonals of **T**, **XGX**^T^, **XPX**^T^, and σ_e_^2^**I**, respectively. Estimates of heritability are obtained from the relevant variances. The off-diagonals of the n × n matrices are the covariances (or correlations if standardized) between the ages. Note that although a linear polynomial is fitted, the graphs of the variances against age are quadratic, because equation (1) is quadratic in age.

#### QTL

The above model was then extended to include an additional term for an age-dependent QTL effect. The model is therefore

y_ij _= μ + (a_i1 _+ a_i2 _× age*) + (c_i1 _+ c_i2 _× age*) + (l_i1 _+ l_i2 _× age*) + f_i _+ e_ij_,

where the terms l_i1 _and l_i2 _are the terms of the linear polynomial linking mean corrected age (age*) to QTL effect. The QTL effect is assumed to have an unstructured variance-covariance structure, with matrix **Q **(with entries q_ij_). The full decomposition, allowing one to calculate estimates of QTL-specific heritabilities is therefore,

**T **= **XGX**^T ^+ **XPX**^T ^+ **XQX**^T ^+ σ_e_^2^**I**

## Results

### Univariate analyses

#### Polygenic

**Figure 1:**
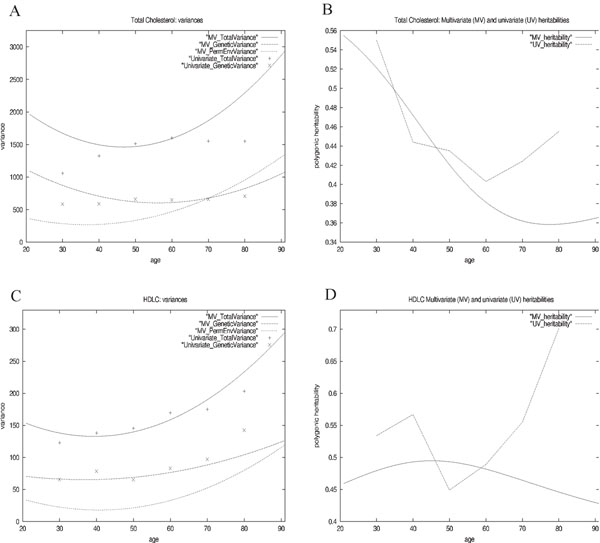
Total cholesterol variances (A) and heritabilities (B), HDLC variances (C) and heritabilities (D)

The results for total cholesterol and HDLC from the ASREML polygenic analyses are superimposed on the multivariate graphs (Figure 1).

#### QTL

A summary of the highest univariate LOD scores is given in Table 2. Note that these LODs have not been corrected for testing multiple trait definitions.

### Longitudinal analysis

#### Polygenic

The longitudinal analyses results for total cholesterol and HDLC are displayed in Figure 1. The results are displayed in two ways. For each trait the variances are shown alongside the variances from the univariate polygenic analyses. Also shown are the heritabilities with the univariate results again superimposed on the same graphs. The correspondence between the univariate and multivariate results is good, particularly in the middle age range (40 to 70). The curves are significantly less accurate for extreme ages because most individuals only have records for ages 40 to 70. While the low order polynomials do not allow the multivariate analyses to closely approximate the univariate heritabilities for traits such as height and HDLC, the true relationship between these traits is likely to be simple, with the univariate results exhibiting stochastic variation around a true smooth curve. Pletcher and Geyer [4] discuss why biological processes will often yield reasonably smooth curves. Table 3 gives the correlations between the traits at different ages. With the exception of BMI, all traits exhibit high genetic correlations across large time periods.

#### QTL

**Figure 2:**
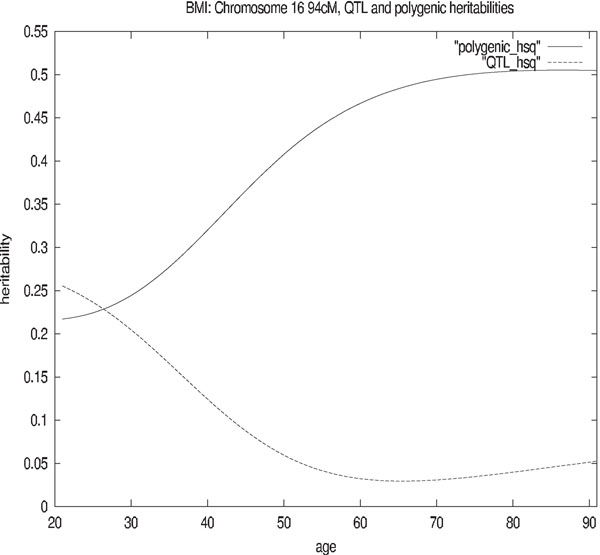
BMI QTL

**Figure 3:**
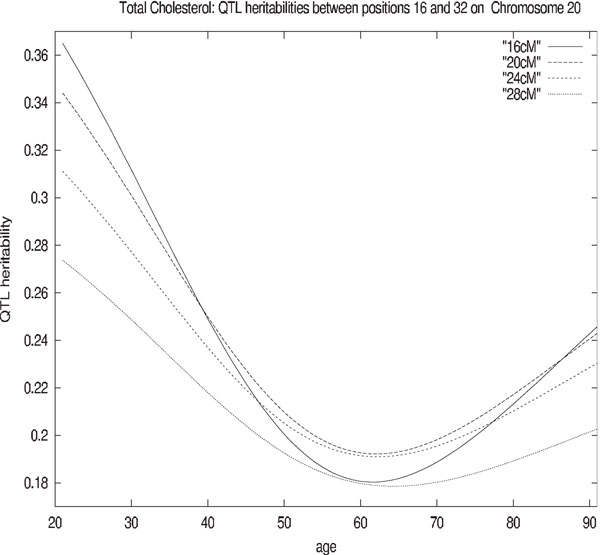
Total Cholesterol QTL

We did not perform a full genome scan of the data. Instead a few of the QTL peaks indicated in the univariate analyses were investigated further. First, the chromosome 16 peak indicated in the univariate analyses was investigated. Figure 2 shows the estimated QTL and polygenic heritability over a range of ages. This QTL is important at lower ages but becomes less so as subjects aged. The correlation between the QTL heritability at age 30 and at age 50 is high (0.86) but falls away more rapidly when one considers ages 50 and 70 (0.48) and ages 30 and 70 (-0.04). Second, the chromosome 20 peak was examined. Figure 3 shows the change in the QTL heritability across chromosome 20. The correlation between the QTL effect at different ages was rather higher than for the chromosome 16 QTL, with the correlation between ages 30 and 70 at 24 cM being 0.45. This QTL accounted for a sizeable proportion of the variance across the range of ages. Third, the peak on chromosome 12 was considered. This QTL explained 5% of the total variation at age 30, with the effect rising to 20% at age 80. The correlation between the QTL effect at ages when the effect was largest was high (0.94 between ages 50 and 70), with it decreasing for ages for which there was less of a QTL effect (0.60 between ages 30 and 50).

We also looked at the other four QTL peaks listed in the univariate results section. However, convergence problems prevented us from obtaining reliable results. Similar problems arose when fitting higher order polynomials to the data.

## Discussion

We performed analyses that explain how the components of variance change over time. The RR model fitted is typically only used for polygenic genetic effects in animal breeding sire models. We have expanded the basic RR model to allow the analyses of both extended pedigrees and marker-specific IBD information. The agreement between the univariate and multivariate analyses performed was good and some of the larger QTL effects were more fully characterized in the longitudinal analyses.

Fitting a higher order polynomial for the relationship between age and the genetic effects may have resulted in a closer fit between the univariate and multivariate results but, in addition to the practical problems of fitting such models, the true relationship between the traits and age is unlikely to be especially complex.

As an alternative to polynomial-based RR approaches, character process models [3] may be useful for longitudinal data analyses, particularly when the correlation between trait measures at distant ages is low. However, when the correlations between trait measures over time is high (as is the case for most of the traits here) polynomial-based methods are effective [3].

The multivariate QTL analyses indicated that one of the QTL detected acted across the range of ages while the other two acted more strongly at the extremes of the age ranges. For some traits there may be correlations between trait value and survival. This may lead to biased QTL effects for QTL acting at later ages. However, maximum likelihood procedures can account for this form of "selection" under certain circumstances [5] and the selection pressure on a single QTL is likely to be small so that a bias in (co)variance estimates may be negligible.

Time constraints prevented a full longitudinal genome scan for QTL but the results shown here indicate that this may be a possibility for other large data sets. The method presented here allows all of the available data to be used in a single powerful analysis.

## Figures and Tables

**Table 1:** Age stratified data. Age bands used for univariate analyses. The multivariate analyses use all the data simultaneously.

Age	20–30	30–40	40–50	50–60	60–70	70–80
Number of individuals	783	1817	2263	1964	1410	879

**Table 2:** Univariate LOD scores

Chromosome	Position (cM)	Trait	Age band for trait	LOD
16	95	BMI	20–30	3.12
5	183	Height	60–70	2.61
10	23	HDLC	70–80	2.50
12	119	HDLC	20–30	2.46
14	138	T. Chol	50–60	2.57
19	101	T. Chol	50–60	3.11
20	24	T. Chol	40–60	3.03

**Table 3:** Phenotypic and Genotypic Correlations. Polygenic model correlations derived from the full longitudinal analyses (Equation 2).

	Phenotypic correlations			Genotypic correlations		
		
Trait	Age 30–70	Age 30–50	Age 50–70	Age 30–70	Age 30–50	Age 50–70
Height	0.79	0.90	0.89	0.83	0.96	0.95
BMI	0.42	0.70	0.84	0.42	0.75	0.91
Total Cholesterol	0.37	0.57	0.61	0.60	0.90	0.88
HDLC	0.41	0.56	0.64	0.80	0.94	0.96

## References

[B1] Gilmour AR, Thompson R, Cullis BR, Welham SJ (2002). ASREML Manual. New South Wales, Department of Agriculture, Orange, 2800, Australia.

[B2] Almasy L, Blangero J (1998). Multipoint quantitative-trait linkage analysisin general pedigrees. Am J Hum Genet.

[B3] Jaffrezic F, Pletcher SD (2000). Statistical models for estimating the genetic basis of repeated measures and other function-valued traits. Genetics.

[B4] Pletcher SD, Geyer CJ (1999). The genetic analysis of age-dependent traits:modeling a character process. Genetics.

[B5] Lynch M, Walsh B (1998). Genetics and the Analysis of Quantitative Traits. Sunderland, MA, Sinauer Associates.

